# Transport of cytotoxic cyanine dyes coupled to siRNA into hematopoietic cancer cells with CD20-, CD22- or CD33-targeting ELART nanocarriers

**DOI:** 10.1038/s41598-026-66482-5

**Published:** 2026-08-18

**Authors:** Timo Krassuski, Nicole Bäumer, Stephan Niland, Katharina Grunert, Lisa Wittmann, Johannes A. Eble, Georg Lenz, Wolfgang E. Berdel, Sebastian Bäumer

**Affiliations:** 1 Department of Medicine A, Hematology, Oncology and Pneumology, University Hospital of Muenster, Albert-Schweitzer-Campus 1, 48149 Muenster, Germany; 2 Institute for Physiological Chemistry and Pathobiochemistry, University of Münster, Waldeyerstraße 15, 48149 Münster, Germany

**Keywords:** Cy5, siRNA-delivery system, Antibody-drug conjugate, Cyanine-dye, Protamine nanocarriers, Biotechnology, Cancer, Cell biology, Drug discovery, Nanoscience and technology

## Abstract

The efficient and safe transfection of hematopoietic cells is a major hurdle that limits efficacy of therapeutic approaches like RNA-interference. We therefore used our modular *EL*ectrostatic *A*ntibody si*R*NA *T*argeted therapy platform (ELART) to develop nanocarriers decorated with antibodies for delivery of siRNA into hematopoietic CD20-, CD22-, or CD33-positive cells. To analyze internalization efficacy into tumor cells, we applied nanocarriers loaded with Cy3- or Cy5-labeled siRNA and reached nearly all target cells within 1–4 h. Exposure to Cy5-labeled non-functional siRNAs resulted in reduction of the mitochondrial membrane potential and reduced viability, as detected in tetramethylrhodamine methyl ester (TMRM) and CellTiter-Glo (CTG) assays. We concluded that with our modular nanocarrier system, we can transport cytotoxic agents such as cyanine dyes when bound to siRNA, as ELART nanocarriers safely complex anionic siRNA electrostatically and releases it intracellularly within the target cell. This proof-of-concept study shows that ELART nanocarriers can transport anti-cancer agents utilizing siRNA as carrier substance.

**Supplementary Information:**

The online version contains supplementary material available at 10.1038/s41598-026-66482-5.

## Introduction

siRNA delivery into cells is limited by its poor stability in biological fluids, inefficient cellular uptake due to its size and negative charge, and rapid clearance or degradation. Even when internalized, siRNA often becomes trapped in endosomes and fails to reach the cytoplasm, while delivery systems can cause toxicity, immune activation, and off-target gene silencing. Achieving efficient, safe, and cell-specific delivery therefore remains the biggest obstacle to the clinical translation of siRNA therapeutics^1–3^. While siRNA transfection *in vitro* is generally part of everyday lab work, especially with adherent cell lines, transfection of non-adherent hematopoietic cells *in vitro* is challenging, as chemical transfection methods usually result in very low transfection efficiency, while harsh methods such as electroporation are associated with low viability and are unsuitable for *in vivo* treatment^4^. To this end, we developed a robust delivery system that efficiently internalizes nucleic acids, and siRNAs in particular, into hematopoietic cells. We already showed for different cell types that ELART nanocarriers composed of antibody-coupled protamine, free protamine and an anionic component such as siRNA spontaneously self-assemble in aqueous solutions and internalize specifically into the intended target cell via antibody-mediated receptor internalization^5–8^. To achieve this, an internalizing cancer-cell specific antibody is chemically conjugated via the bifunctional linker sulfo-SMCC to the carrier protein protamine, which is a natural DNA-condensing sperm protein^9^. Together with unbound protamine, oncogene specific siRNA is electrostatically complexed, and these three components form a nanocarrier. Since this modular system can be applied (1) to any cell surface protein to determine internalization to the target cell and (2) to any gain-of-function factor by designing the appropriate siRNA, we already showed efficacy of a nanocarrier targeting KRAS-mRNA in EGFR-positive solid tumors and a nanocarrier targeting EWS-FLI1 translocation product in Ewing sarcoma cells^6^. The high medical need but also the technical limitations of siRNA transport into hematopoietic cells challenged us to apply our nanocarrier system to cells of hematopoietic origin.

We used the well-established anti-CD20-antibody rituximab, anti-CD22-antibody inotuzumab and anti-CD33-antibody gemtuzumab and formulated above described targeted nanocarriers with those. We then performed an in-depth characterization, starting with internalization efficacy into the target cells. As observed previously, commercial Alexa488-siRNA revealed high internalization efficiency when analyzed by flow cytometry^6^, but only a small population seemed to be successfully transfected, as assessed by microscopic analysis^5,6^. To optimize microscopical analyses of siRNA transfection, we switched to cyanine-dye Cy3- and Cy5-labeled siRNAs which are a usual technical tool to visualize siRNAs.

Cyanine dyes are a class of synthetic fluorescent molecules that have attracted interest as anti-cancer agents as some cyanine dyes showed preferential accumulation in tumor cells, which is attributable to the elevated mitochondrial membrane potential, the uptake of cyanine-albumin aggregates via the enhanced permeability and retention (EPR) effect and in some cases to the uptake via the overexpression of organic anion transporter peptides (OATP)^10–12^. Many cyanine dyes exert anti-cancer effects by disrupting mitochondrial function, generating reactive oxygen species, or acting as photosensitizers in photodynamic and photothermal therapy, leading to selective cancer cell death^13^. Their intrinsic imaging capability also enables theranostic applications^14^, although challenges such as dark toxicity, photobleaching, and limited *in vivo* stability remain barriers to clinical translation. In addition to their phototherapy roles, some cyanine derivatives show direct antiproliferative effects against cancer cells *in vitro*: Penetrating cells, interacting with intracellular targets (e.g., topoisomerase II), and inducing cytotoxicity with selectivity against certain tumor lines such as colorectal cancer^15^. As often, a cell-specific uptake of cyanines is a general problem and many attempts are performed to improve cyanine transport into cancer cells^16–18^.

To solve these targeting problems, we propose the packaging of cyanine-labeled siRNA into ELART nanocarriers. We show here that cyanine-dye labeled siRNAs are (1) efficiently bound in ELART nanocarriers, (2) transported in CD20-, CD22- or CD33-positive cells, respectively, and (3) inhibit growth of target cells. This concept can serve as a blueprint for electrostatic binding of other chemical components via the linker siRNA to the cationic carrier protein protamine within the ELART nanocarrier. Within a broader scope, generally toxic drugs could be administered in a targeted, modular manner, thereby losing some of their toxicity and adverse side-effects outside the intended target cells.

## Results

**Fig. 1:**
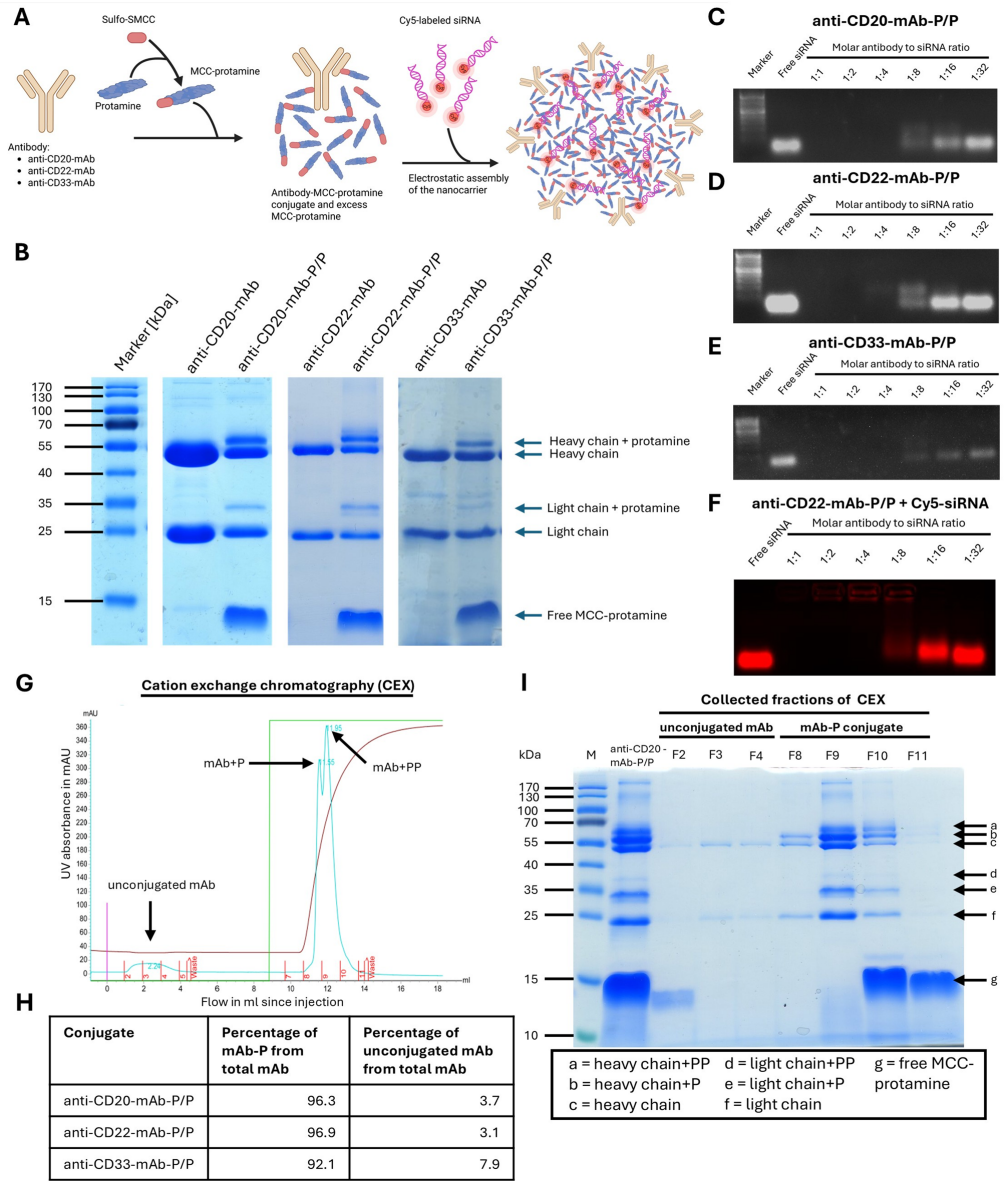
Assembly of anti-CD20-mAb-P/P, anti-CD22-mAb-P/P and anti-CD33-mAb-P/P nanocarriers.(**A**) Schematic illustration of the nanocarrier assembly process. The targeting antibody is conjugated to the cationic nucleic acid binding protein protamine using the crosslinker Sulfo-SMCC. This yields a mixture of antibody-protamine conjugates and excess free MCC-protamine which can complex labeled-siRNA via electrostatic binding, resulting in spherical nanocarriers. (**B**) PAGE analysis of anti-CD20-mAb-P/P, anti-CD22-mAb-P/P and anti-CD33-mAb-P/P in comparison to unconjugated antibody. Successful conjugation was visible by a size shift of the light and heavy chain due to the addition of protamine. Gel images were cropped to enhance visual clarity. The corresponding uncropped original gel images are provided in Fig. S2A (**C-E**) Band shift assays via agarose gel electrophoresis for the binding capacity of anti-CD20-mAb-P/P (**C**), anti-CD22-mAb-P/P (**D**) and anti-CD33-mAb-P/P (**E**). The mAb-P/P conjugate was complexed with siRNA in molar ratios between 1:1 and 1:32 and as soon as the binding capacity of the conjugate was surpassed, a siRNA band reappeared in the gel. (**F**) Agarose-gel electrophoresis band shift assay for the complexation of Cy5-labeled siRNA with anti-CD22-mAb-P/P. Comparable charge extinction ratios (about 8 mol siRNA/mol of conjugate) showed that complexation of siRNA is not influenced by the addition of Cy5 to the siRNA. The corresponding uncropped gel images for C-F. are provided in Fig. S2B (**G**) Chromatogram of the cation exchange (CEX) chromatography of anti-CD20-mAb-P/P (for the anti-CD22- and anti-CD33-mAb-P/P conjugates see Fig. S1). UV absorbance is shown in blue, conductivity in brown and fraction pump B in green. A small peak was evident in the flow-through fraction, representing the unconjugated mAb, whereas the elution peak exhibited two maxima corresponding to mono- and bi-conjugated mAbs. (**H**) Percentage of conjugated and unconjugated mAbs for anti-CD20-, anti-CD22- and anti-CD33-mAb-P/P as determined by CEX chromatography. (**I)** PAGE analysis of the CEX chromatography of anti-CD20-mAb-P/P shown in G. In the flow-through fraction, only faint bands for unconjugated mAb were present, whereas prominent bands corresponding to protamine-conjugated mAbs were evident in the elution fraction. The uncropped gel image is provided in Fig. S2C.

We previously developed a nanocarrier platform for the directed transport of siRNA into tumor cells via receptor mediated uptake, called ELART (*EL*ectrostatic *A*ntibody si*R*NA *T*argeted therapy) nanocarriers. Initially, we used Cy5-labeled siRNA conjugates to track the internalization efficiency into cells *in vitro* and *in vivo* but soon realized that we can also exploit the tumor targeting effect of our nanocarriers to safely complex cytotoxic substances such as cyanine dyes and deliver them to tumor cells using siRNA merely as a carrier substance. To assemble an ELART nanocarrier (Fig. 1A), the natural nucleic acid binding protein protamine is conjugated with a 5-fold molar excess of the crosslinker sulfo-SMCC to yield MCC-protamine. This activated maleimide MCC-protamine is thiol-conjugated to cysteines of the targeting antibody, in this case directed against CD20, CD22 or CD33, in a 32:1 molar ratio and generates an antibody-MCC-protamine conjugate (mAb-P). This conjugate, together with excess MCC-protamine from the coupling reaction (referred to as mAb-P/P), assembles electrostatically into spherical nanocarriers upon addition of siRNA, either unlabeled or labeled with a compound like Cy3 or Cy5 (Fig. 1A). Polyacrylamide gel electrophoresis (PAGE) analysis of anti-CD20-mAb-P/P, anti-CD22-mAb-P/P and anti-CD33-mAb-P/P revealed that all conjugates exhibited a size shift in both the light and heavy chains, indicating successful addition of protamine to the antibody (Fig. 1B). Additionally, a prominent band of free MCC-protamine was visible at the bottom of the gel, which is required for nanocarrier assembly (Fig. 1B). The siRNA binding capacity of this mAb-P/P conjugate was determined by a band shift assay, and a binding capacity of just under 8 siRNAs per antibody was achieved irrespective of the targeting antibody (Fig. 1C-E). This binding capacity remained unchanged when Cy5-labeled siRNA was used (Fig. 1F). The conjugation efficacy of the respective mAb to protamine by means of bispecific crosslinker sulfo-SMCC was determined by cation exchange (CEX) chromatography and found to be between 96.9 and 92.1% depending on the antibody conjugate (Figs. 1G-H and S1). PAGE analysis of CEX fractions revealed addition of up to two protamine molecules on the heavy and light chain indicated by two shifted bands above the corresponding heavy or light chain band (Figs. 1I and S1).

**Fig. 2:**
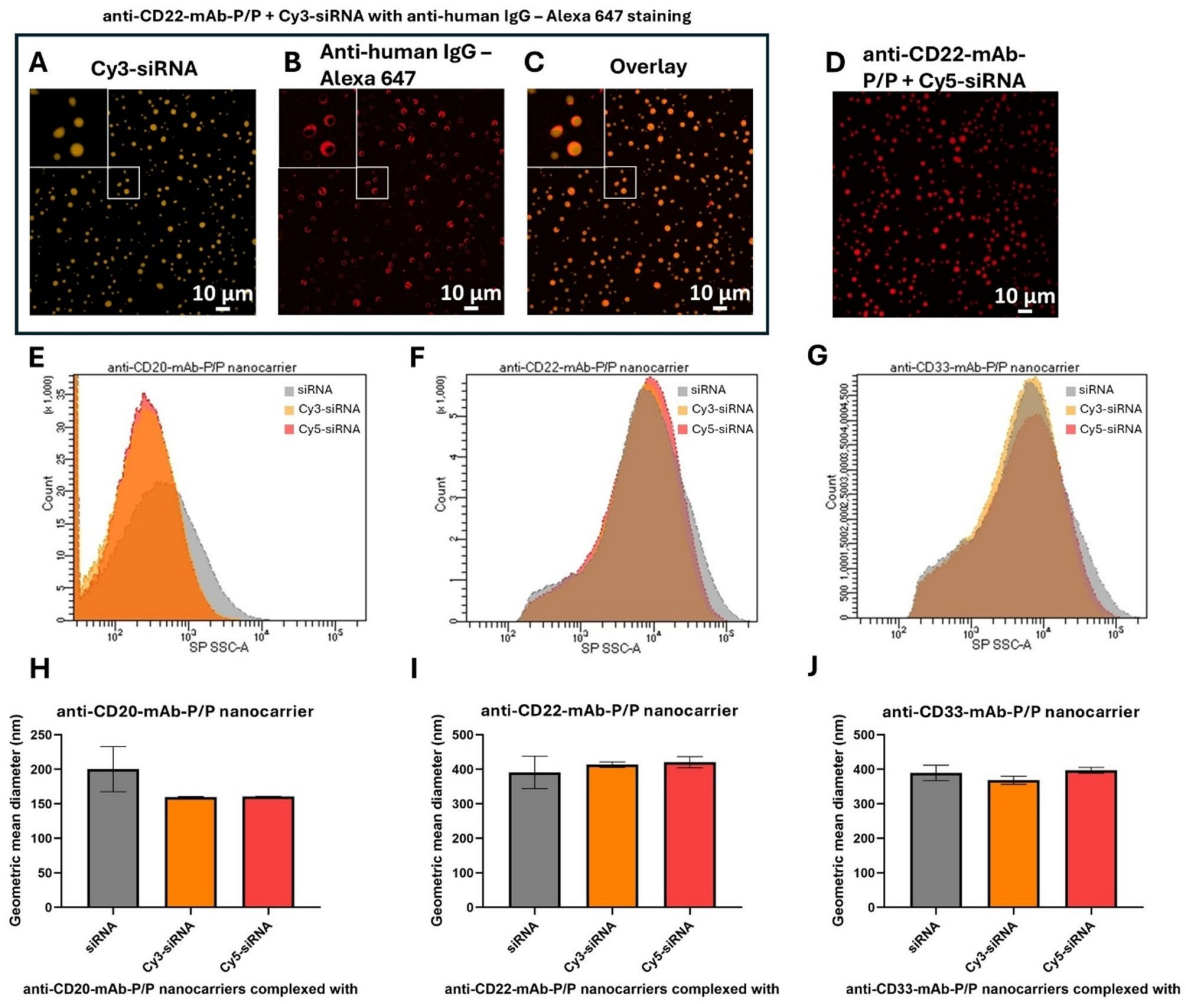
Nanocarrier structure and size distribution. (**A-C**) LSM images of anti-CD22-mAb-P/P+Cy3-siRNA nanocarriers stained with anti-human-IgG-Alexa 647. (**A**) Cy3-siRNA. (**B**) anti-human-IgG-Alexa 647. (**C**) Overlay. (**D**) Anti-CD22-mAb-P/P+Cy5-siRNA nanocarriers exhibit a similar morphology as Cy3-siRNA complexed carriers in A. (**E-G**) Small particle side scatter area (SP-SSC-A) histograms of anti-CD20-mAb-P/P (**E**), anti-CD22-mAb-P/P (**F**) and anti-CD33-mAb-P/P (**G**) nanocarriers complexed with siRNA, Cy3-siRNA and Cy5-siRNA. The addition of a cyanine dye to the siRNA does not significantly influence the size distribution of nanocarriers. (**H-J**) Geometric mean sizes of anti-CD20-mAb-P/P (**H**), anti-CD22-mAb-P/P (**I**) and anti-CD33-mAb-P/P (**J**) nanocarriers complexed with siRNA, Cy3-siRNA and Cy5-siRNA. Addition of a cyanine dye to the siRNA does not significantly influence the size of the respective nanocarrier (one way ANOVA, not significant). Nanocarriers were complexed three times independently per condition. Size determination was performed by flow cytometry using size reference beads (Fig. S3A-B).

To assess availability of the targeting antibody on the surface, we assembled anti-CD22-mAb-P/P nanocarriers with Cy3-siRNA and stained those with an anti-human IgG-Alexa-647 antibody. The Cy3-siRNA fluorescence was confined in the core of the carrier while antibody staining resulted in ring-like structures around the perimeter of the carrier, implying structural availability of the antibody for directed targeting (Fig. 2A-C). Microscopically, we found no morphological difference of nanocarriers complexed with Cy3-siRNA to those complexed with Cy5-siRNA, as both resulted in particles with a similar size distribution and homogeneously round particles (Fig. 2A, D). Nanocarriers assembled with unmodified siRNA and Cy3- or Cy5-siRNA were analyzed by flow cytometry and evidently addition of Cy3 or Cy5 to the siRNA did not have a strong effect on the small particle side scatter area (SP-SSC-A) distribution of nanocarriers (Fig. 2E-G). To determine the approximate sizes of those nanocarriers we performed a size calibration using size reference beads, thereby converting the SP-SSC-A to diameter (see Fig. S3 A-B) and calculated the geometric mean size of the nanocarrier distribution. Size was dependent on the antibody used in the conjugate with anti-CD20 nanocarriers having geometric means of 160–200 nm while anti-CD22 or anti-CD33 nanocarriers were 370–420 nm in size (Fig. 2H-J). Addition of Cy3 or Cy5 to the siRNA did not have a significant effect on size (Fig. 2H-J). We also found fluorescence of Cy3 or Cy5 containing nanocarriers to be directly proportional to their size indicating a fixed ratio of siRNA and conjugate (Fig. S3 C-H).

**Fig. 3:**
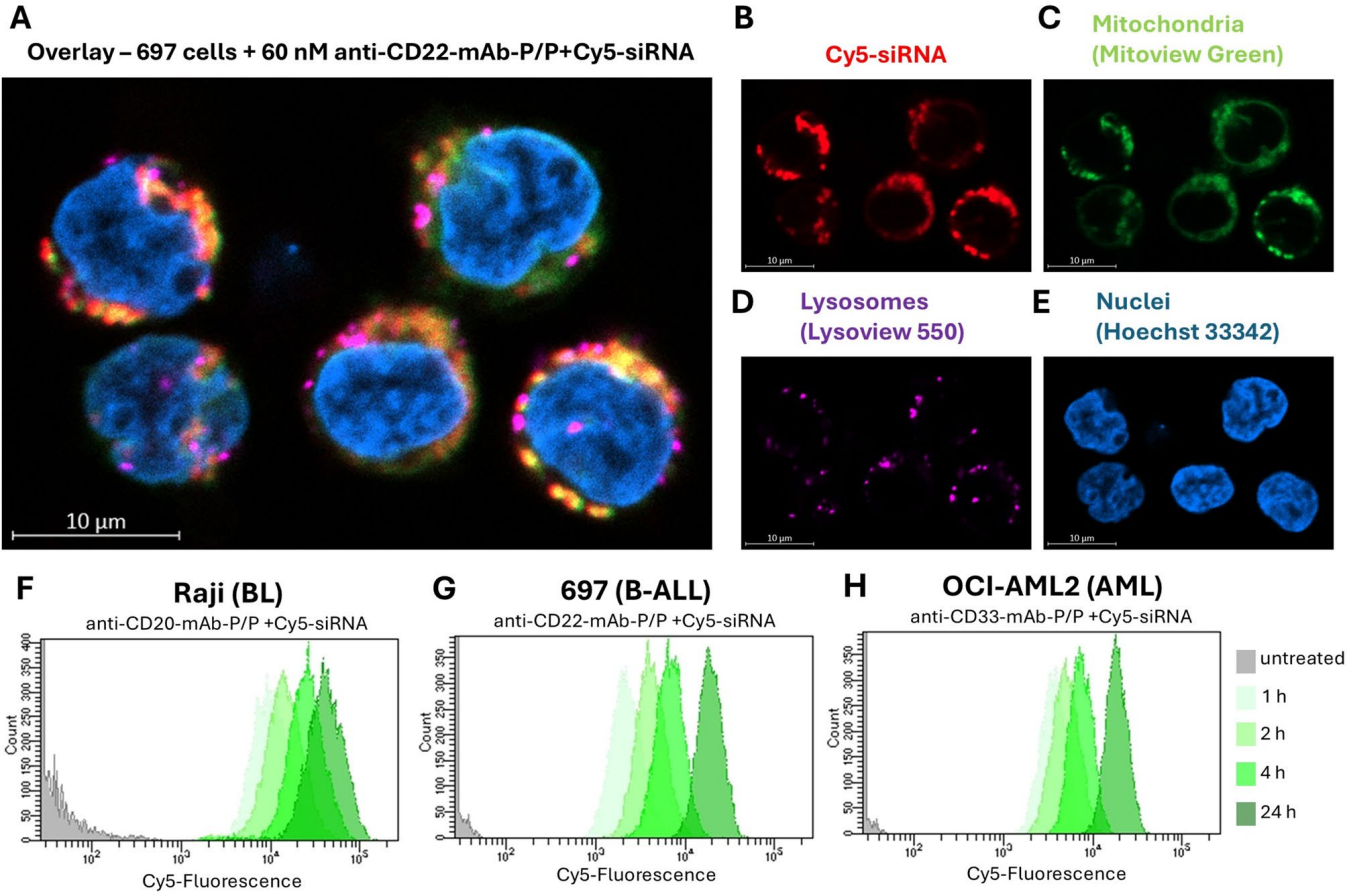
Internalization of nanocarriers into tumor cells. Intracellular localization of the Cy5-siRNA 24 h after treatment of 697 cells with 60 nM anti-CD22-mAb-P/P+Cy5-siRNA determined by laser scanning microscopy. A complete colocalization of Cy5 signal and mitochondria was evident as shown by the yellow signal in the overlay (**A**). Cy5-siRNA (red) (**B**), mitochondria (green) (**C**), lysosomes (purple) (**D**) and nuclei (blue) (**E**) were found in distinct locations. (**F-H**) Flow cytometric analysis of the uptake of Cy5-siRNA containing nanocarriers into target cells. Cells were incubated with the carriers for 1 to 24 h. (**F**) Internalization of anti-CD20-mAb-P/P+Cy5-siRNA nanocarriers into Raji cells. (**G**) Internalization of anti-CD22-mAb-P/P+Cy5-siRNA nanocarriers into 697 cells. (**H**) Internalization of anti-CD33-mAb-P/P+Cy5-siRNA nanocarriers into OCI-AML-2 cells. All cells were Cy5-positive after 1 h of incubation with fluorescence intensity increasing further until 24 h (increasing green intensity).

To assess uptake and subcellular localization of the Cy5-siRNA delivered via anti-CD22-mAb-P/P nanocarriers the B-ALL cell line 697 was treated with 60 nM anti-CD22-mAb-P/P + Cy5-siRNA nanocarriers and analyzed for subcellular localization of the Cy5-fluorescence after 24 h via laser scanning microscopy (LSM). The Cy5-fluorescence was colocalized with the mitochondria, while little to no colocalization with lysosomes was observed (Fig. 3A-E). To analyze the time dependence of nanocarrier uptake, we selected Raji (BL), 697 (B-ALL) and OCI-AML2 (AML) cells to be targeted with anti-CD20, anti-CD22 or anti-CD33 nanocarriers, respectively. Cells were incubated with 60 nM of Cy5-siRNA containing nanocarriers (referring to the respective antibody) for 1–24 h and Cy5-fluorescence was measured by flow cytometry. Within 1 h of incubation all cells were Cy5 positive, while Cy5-fluorescence increased further with prolonged incubation times (Fig. 3F-H). This was similar for all cell lines and nanocarrier formulations tested.

**Fig. 4:**
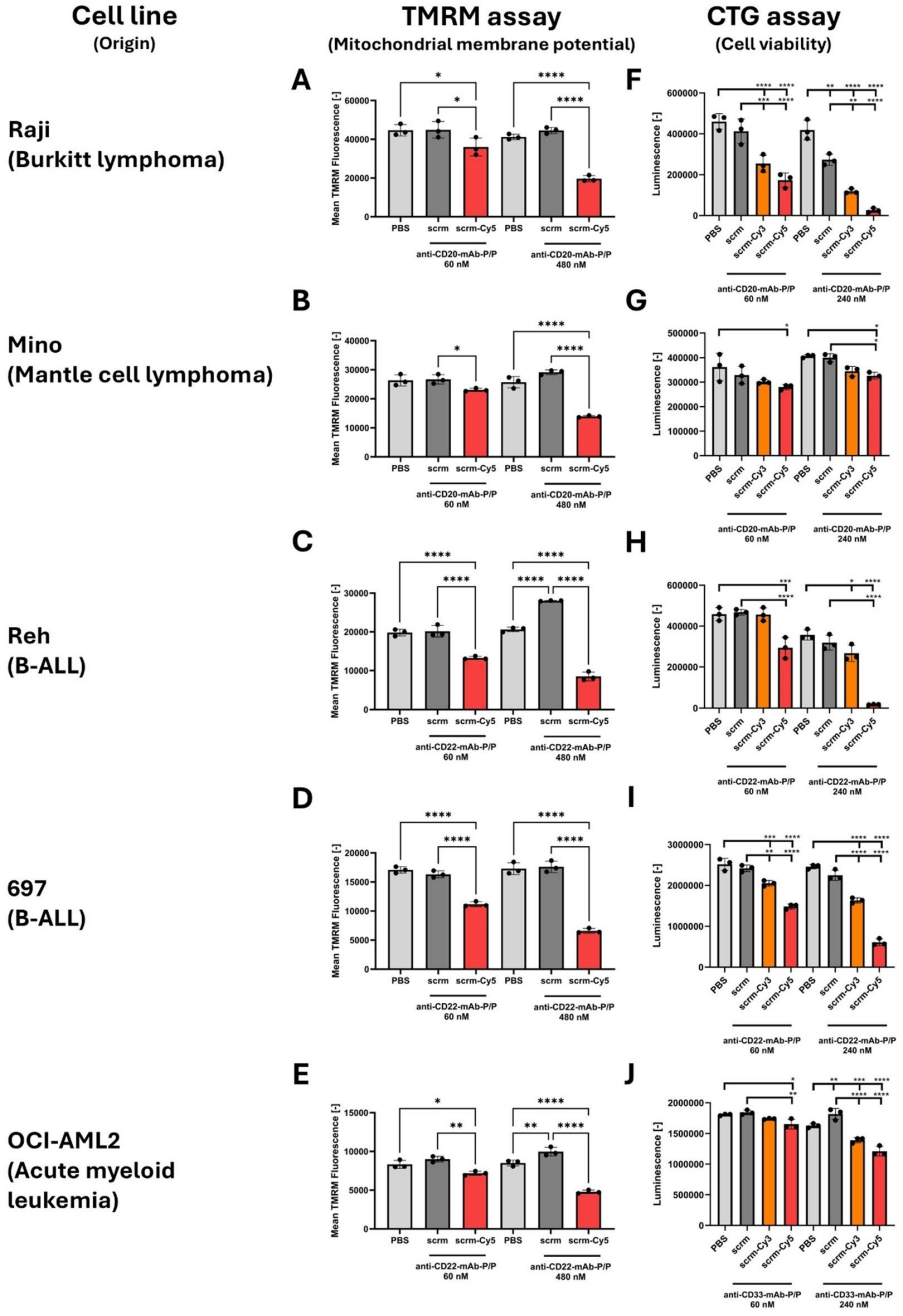
Treatment of lymphoma and leukemia cells with nanocarriers transporting Cy5-siRNA reduces the mitochondrial membrane potential and impairs proliferation and viability. (**A-E**)TMRM assay to measure the mitochondria membrane potential showed strong reduction of the mitochondrial membrane potential upon treatment with Cy5-siRNA. Cells were treated with 60 and 480 nM nanocarriers formulated with either scrm-siRNA or scrm-Cy5-siRNA in comparison to cells treated with a solvent control (PBS). Raji and Mino cell were treated with anti-CD20-mAb-P/P nanocarriers (**A-B**), Reh and 697 cells with anti-CD22-mAb-P/P nanocarriers (**C-D**) and OCI-AML2 cells with anti-CD33-mAb-P/P nanocarriers (**E**). (**F-J**) CTG assays of the cell lines from A-E to determine the effect of nanocarrier treatment with Cy3 or Cy5 siRNA on proliferation and viability at 60 and 240 nM nanocarrier concentration. Cy5-siRNA significantly reduced the luminescence signal, which is proportional to viable cell count and thus decreased proliferation and viability in treated cells. Cy3-siRNA was less effective across all cell lines. Experiments were performed in biological triplicates and CTG assays additional with three technical replicates. Statistics for all TMRM or CTG assays were performed with a one-way ANOVA and Sidak’s multiple comparisons test. Error bars represent standard deviation. Significances: *= *p* < 0.05, **= *p* < 0.01, ***= *p* < 0.001, ****= *p* < 0.0001.

Since mitochondrial targeting of Cy5-siRNA was evident, we analyzed the mitochondrial membrane potential by TMRM assay, as accumulation of Cy5 in or on the mitochondria could influence mitochondrial function. Cy3 has a very similar fluorescence spectra as TMRM and could not be measured by this assay. To separate the effect of the Cy5 label from that of siRNA, a scrambled (scrm) siRNA was used, which has no cellular target and therefore no specific effect on cells. A strong reduction of the mitochondrial membrane potential was observed across all cell lines tested, which increased with nanocarrier concentration (Fig. 4A-E). Responsiveness to Cy5-siRNA treatment at 60 nM nanocarriers was dependent on the cell line, while at 480 nM all cell lines exhibited a reduction in the mitochondrial membrane potential of more than 50% (Fig. 4A-E). For two cell lines (Reh and OCI-AML-2 Fig. 4C, E) a significant hyperpolarization of the mitochondrial membrane potential was measured at 480 nM nanocarriers with scrm-siRNA, which is a known effect of protamine sulfate and is attributable to the protein components, independent of the siRNA^19^. As a reduction in mitochondrial membrane potential is a sign of cell stress and can be an early indication of apoptosis, we performed cell titer glow assays to test whether treatment with Cy5- or Cy3-siRNA nanocarriers has an influence on proliferation and viability. Across all cell lines tested an antiproliferative effect of treatment with Cy5-siRNA nanocarriers was evident, especially at higher nanocarrier concentrations (Fig. 4F-J). The degree of signal reduction depended on the cell line, with Mino and OCI-AML-2 cells showing only a slight reduction in signal after treatment, while Raji, 697, and Reh cells presented a strong antiproliferative effect, particularly at nanocarrier concentrations of 240 nM. The antiproliferative effect of Cy3-siRNA was less pronounced than that of Cy5-siRNA, but at high concentrations it was able to significantly reduce the number of viable cells in most cell lines.

**Fig. 5:**
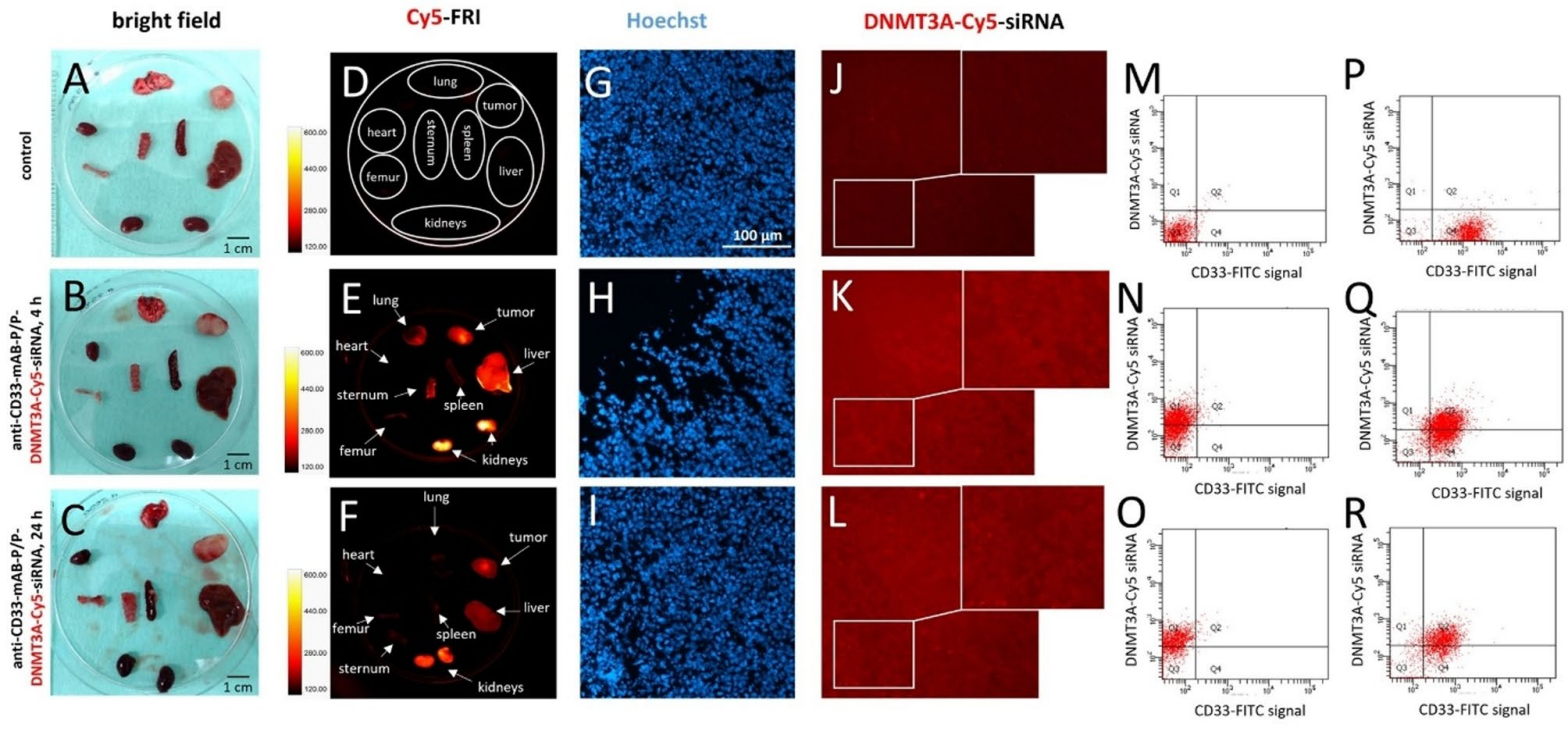
Biodistribution of anti-CD33-mAb-P/P-DNMT3A-Cy5-siRNA nanocarriers. (**A-C**) Bright field images of prepared organs of an untreated control mouse (**A**), representative mice sacrificed 4 h after anti-CD33-mAb-P/P-DNMT3A-Cy5-siRNA treatment (**B**) and representative mice sacrificed 24 h after anti-CD33-mAb-P/P-DNMT3A-Cy5-siRNA treatment (**C**). (**D-F**) Cy5-FRI images of organs shown in A-C. Cy5-fluorescence in treatment groups is predominantly found in tumors, livers and kidneys. Approximate location of the organs is schematically shown in D as organs from the control mouse were non-fluorescent. Organs are similarly arranged across A-F. (**G-I**) Hoechst staining of tumor sections from the tumors shown in A-C. The scale bar is representative for images G-L. (**J-L**) Cy5-fluorescence image of the tumor sections shown in G-I. An increase in Cy5-fluorescence is visible in treatment groups compared to untreated control. (**M-R**) Flowcytometric analysis of isolated cells from leukemic tumors shown in A-C. Tumor cells were either analyzed unstained (**M**-**O**) or stained with an anti-CD33-FITC antibody (**P**-**R**). Treated tumors exhibited an increase in Cy5-fluorescence and a reduction in accessible CD33 on the surface. Gating strategy: LL: Cy5 negative and CD33-FITC negative. LR: Cy5 negative and CD33-FITC positive. UL: Cy5 positive and CD33-FITC negative. UR: Cy5 positive and CD33-FITC positive.

A prerequisite for systemic treatment of cancer cells with cytotoxic substances is specific tumor targeting. To get a deeper insight into the biodistribution of Cy5-siRNA containing nanocarriers, OCI AML2 cells were subcutaneously xenografted into CD1 nude mice and, as soon as the tumor reached a size of 800–1100 mm^3^, treated with 4 mg/kg body weight anti-CD33-mAb-P/P+DNMT3A-Cy5-siRNA via intraperitoneal injection. Mice were sacrificed 4–24 h after treatment and extracted organs were analyzed for Cy5-fluorescence. Instead of scrm-Cy5-siRNA, DNMT3A-Cy5-siRNA was used, as we had already conducted a successful biodistribution experiment with this siRNA in a similar experiment^5^. Analysis of Cy5-fluorescence in the organs via fluorescence reflectance imaging (FRI) 4 h after treatment revealed accumulation of the DNMT3A-Cy5-siRNA in the leukemic tumor, liver and kidneys, as well as in small amounts in the lung and sternum (Fig. 5E). 24 h after treatment Cy5-fluorescence was detectable in the leukemic tumor, liver and kidneys (Fig. 5F). Sections of extracted leukemic tumors revealed increased Cy5-fluorescence in the treated tumor tissues compared to untreated tumors (Fig. 5J-L). Hoechst staining of the tumor sections are shown next to respective Cy5-fluorescence pictures (Fig. 5G-I). Isolated tumor cells from the tumor tissue were analyzed by flow cytometry and tumor cells from treated tumors exhibited an elevated Cy5-fluorescence compared to an untreated tumor (Fig. 5M-O). Staining of tumor cells with an anti-CD33-FITC antibody that binds to a different epitope than the nanocarrier-bound CD33 targeting mAb revealed a decrease in accessible CD33 on the surface of the cells indicating successful uptake of Cy5-siRNA-containing anti-CD33-mAb-P/P nanocarriers via receptor-mediated internalization (Fig. 5P-R).

## Discussion

Intensive research is still being conducted into the effective treatment of cells with siRNA, especially *in vivo*. *In vitro*, even the transfection of suspension cells such as leukemic and lymphatic cell lines still poses problems. There are high hopes for RNA interference as a highly specific therapeutic option in gain-of-function diseases such as cancer, viral infections and neurological diseases, because siRNA can inhibit the mRNA of gain-of-function proteins and thus bring the corresponding disease under control. When the necessary siRNA transport is combined with a cell type-specific delivery system such as the ELART nanocarrier, this results in a modular, efficient and safe therapeutic option.

Here, we present ELART nanocarriers to target CD20-, CD22- and CD33-positive cells, respectively. While we have previously shown transport of anionic ibrutinib-Cy3.5 in anti-CD20- and anti-CD33-functionalized nanocarriers^5,7^ and also siRNA-transport in anti-CD33-mAb-P/P-nanocarriers^5^, the coupling of the anti-CD22 antibody inotuzumab to protamine is presented here for the first time. Inotuzumab is part of the FDA approved antibody-drug conjugate (ADC) inotuzumab ozogamicin composed of a humanized IgG4 monoclonal antibody that targets CD22, a B-cell surface antigen highly expressed on most B-cell precursor acute lymphoblastic leukemia (B-ALL) cells, linked via an acid-labile linker to the potent cytotoxic agent calicheamicin^20^. As expected, CD22-positive cells internalize our inotuzumab decorated nanocarriers and release Cy5-coupled siRNA in almost all cells. In addition to inotuzumab, we have successfully formulated nanocarriers with clinically approved antibodies from other applications, such as the anti-CD20-mAb rituximab^21^ and the anti-CD33-mAb gemtuzumab presented here, as well as the anti-EGFR antibody cetuximab to target solid tumor cells^6,22–24^ and the anti-IGF_1_R-antibody teprotumumab to target Ewing sarcoma^6^. Therefore, the specific and efficient targeting of ELART nanocarriers can be modularly applied to any cell type, as soon as an internalizing antibody that binds to a specific surface protein is found.

siRNA as an active drug to knock-down an intracellular mRNA is of course a desirable load. We have previously demonstrated successful knockdown of KRAS^6^, PIK3CA^23^, DNMT3A^5^, FLT3-ITD^5^ or EWS-FLI1^6^. For many cancer cells, causative gain-of-function mutations or overexpressed proteins are known, and the cancer cell can become addicted to the respective pathway, which therefore is a good target match for specific therapy. However, silencing one or even two of these factors by RNAi may not be sufficient to eliminate the cancer cell; instead, a broader yet still specific therapy approach may be required. Conventional chemotherapy is a very broad therapy approach which often lacks specific targeting. According to the modular character of the ELART technology, also other anionic molecules such as ibrutinib-Cy3.5 and special photodynamic compounds can be transported within the nanocarriers^5,7,8^. During internalization studies with cyanine-labeled siRNAs, the idea arose that siRNA could be used merely as an anchor for a cytotoxic agent. The transport of this cytotoxic agent would then be much more specific and safer. The cyanine dyes Cy3 and Cy5 were an obvious option for a proof-of-concept study, because siRNAs can readily be ordered linked to them, their uptake can be monitored by fluorescence and induced mitochondrial depolarization can easily be measured. Cy3 or Cy5 are not commonly known to be highly cytotoxic, although cyanine dyes beyond Cy3 and Cy5 may also be realistic therapy options, since many attempts with cyanine compounds especially in the field of photodynamic therapies were already ongoing^25^. For cyanine dyes in photodynamic or photothermal therapy usually a low dark toxicity is required to minimize off target effects in healthy tissue. By improving targeting via efficient and safe complexing in nanocarriers, cyanine dyes with a high dark toxicity could become promising pharmaceutical agents - possibly obviating the necessity of light irradiation. Additionally, the accumulation of cyanine dyes in or on mitochondria poses an additional level of safety, as the cyanine dye is retained in the tumor cell once it is released from the nanocarrier due to the tumor cell’s elevated mitochondrial membrane potential. The coupling of siRNA and cytotoxic agents with potential synergistic effects is not entirely new, as researchers have chemically conjugated doxorubicin (a classic cytotoxic chemotherapy) to siRNA via a disulfide-based self-immolative linker that is cleaved inside the reducing environment of cancer cells. This type of design aims to release both the siRNA and the drug intracellularly to combine gene silencing with cytotoxic effects^26^. Lacking an electrostatic and specific delivery platform such as ELART, quite complex dual siRNA-chemotherapy delivery systems have been investigated^27^, but did not reach clinical stage. Co-packaging of siRNA and chemotherapeutic agents such as docetaxel in classical nanoparticles might face leakiness, which releases the drugs before reaching the target cell^28^. As 5´ modified siRNA is known to retain knockdown efficacy, future studies will show if our design can be used as a combinatory system of functional siRNA and cytotoxic compound with the benefit of cell specific delivery^29,30^.

The ELART nanocarriers as presented here have therefore implications for *in vitro*, experimental *in vivo* and systemic therapeutic applications. Suspension cells might be transfected more efficiently *in vitro*, targeting efficacy is almost 100%, while vitality of cells treated with the carrier with non-fluorescent siRNA is almost 100%. ELART nanocarriers are also very efficient upon systemic application *in vivo* as shown for CD33-nanocarriers here and published for CD20-functionalized nanocarriers^7^. *In vivo* application of the novel CD22-nanocarrier will be part of future studies, especially in combination with functional siRNA against oncogenes. In the treatment of MCL^31^, BL^32^, B-ALL^33,34^ and AML^35^, despite recent advances in therapy options, a substantial unmet medical need for optimal therapy persists, positioning the anti-CD20, anti-CD22 and anti-CD33 nanocarriers presented here as promising candidates. Further studies will show whether the therapeutic options for hematologic malignancies can be expanded by highly specific and efficient delivery of siRNAs either alone or in combination with cytotoxic agents such as cyanine dyes using our nanocarriers.

## Materials and methods

### Chemical conjugation of anti-CD20, anti-CD22 and anti-CD33 monoclonal antibody

The conjugation of mAbs was performed similarly to our previous publications^5,8^. For conjugation of the mAbs, 3.9 mM protamine sulfate (Cat. No. 539122, Calbiochem, Frankfurt a.M., Germany) was coupled to 19.5 mM sulfonsuccinimidyl-4-(N-maleimidomethyl)cyclohexan-1-carboxylate (SMCC; Pierce No. 22622, Thermo Fisher Scientific Inc., Waltham, MA, USA) in pH-adjusted MilliQ-H_2_O (pH of 6.0–7.0 with 0.1 M carbonate buffer (pH 8.3)). After 100 min of shaking incubation at 31 °C, the generated protamine-(N-maleimidomethyl)-cyclohexan-1-carboxylate (protamine-MCC) was purified from excess linker by gel filtration chromatography using HiPrep 26/10 desalting columns (Cat. No. GE17-5087-02, Sigma-Aldrich, St. Louis, MO, USA). The anti-CD22 and anti-CD33 mAbs were purified as described in the corresponding methods section, the anti-CD20 mAb rituximab (Truxima®, Mundipharma) was desalted into PBS using HiPrep 26/10 desalting columns (Cat. No. GE17-5087-02, Sigma-Aldrich, St. Louis, MO, USA). Purified protamine-MCC was coupled to the desalted anti-CD20-, anti-CD22- or anti-CD33-mAb in a 32:1 molar ratio overnight at 4 °C. Excess MCC-protamine that did not react with the antibody remains in the conjugate mixture. For all chromatography steps an Äkta Pure™ 25 (1.2.0.0) system with data acquisition via UNICORN™ 6.3 (all Cytiva, Washington, D.C., USA) was used.

### Electrostatic assembly of nanocarriers

For electrostatic assembly of nanocarriers, the anti-CD20-mAb-P/P, anti-CD22-mAb-P/P or anti-CD33-mAb-P/P, which have a concentration between 6 and 12 µM referring to the antibody portion of the conjugate, were directly mixed with 5-fold molar excess of siRNA (40 µM in nuclease free water or 80 µM in nuclease free water for *in vivo* experiments), mixed vigorously by pipetting and incubated at RT for 1 h. Dilution of the conjugate or siRNA with PBS is not required for complexation within the above stated concentration ranges. After the incubation the nanocarrier formulation was diluted with cell culture medium or PBS to a final concentration of 60, 240 or 480 nM (complexation and dilution volumes are dependent on the intended assay). The sequences of the used siRNAs are as follows:

scrm-siRNA (sense: 5′GGCCGACACCGUCAUUUAAUUdTdT′3; antisense: 5′AAUUAAAUGACGGUGUCG GCCdTdT′3),

scrm-Cy3-siRNA (sense: 5′Cy3-GGCCGACACCGUCAUUUAAUUdTdT′3; antisense: 5′AAUUAAAUGACGGUGU CGGCCdTdT′3 ),

scrm-Cy5-siRNA (5′Cy5-GGCCGACACCGUCAUUUAAUUdTdT′3; antisense: 5′AAUUAAAUGACGGUGUCGG CCdTdT′3).

DNMT3A-Cy5-siRNA (5′Cy5-GAACAGAAGGAGACCAACAdAdA′3; antisense: 5′UGUUGGUCUCCUUCUG UUCdAdA′3).

The scrm-siRNA is a control siRNA that lacks a cellular target and therefore only reflects the effect of the cyanine label. The concentration of nanocarriers always refers to the molarity of antibodies in the assembled carrier.

### Biodistribution

All animal experiments in this study were carried out in accordance with the recommendations of the state office Animal Care and Use Committee ‘‘Landesamt für Natur, Umwelt und Verbraucherschutz NRW” (LANUV). This study was performed with permission of the Institutional Animal Care and Use Committee and of the local veterinary administration of Muenster (Permit Number 81-02.04.2019.A341). Prior to analysis, CD1 nude mice (sourced from Charles River Laboratories, Sulzfeld, Germany) were deeply anesthetized with CO_2_ and euthanized by cervical dislocation. For experimental details, see the supplementary methods.

## Supplementary Information

Below is the link to the electronic supplementary material.

Supplementary Material 1

## Data Availability

The datasets generated during the current study are available from the corresponding author on reasonable request.

## Abbreviations

ELART: Electrostatic antibody siRNA targeted therapy
siRNA: Small interfering RNA
TMRM: Tetramethylrhodamine methyl ester
CTG: Cell titer glow
EPR: Enhanced permeability and retention
OATP: Organic anion transporter peptides
B-ALL: B-cell acute lymphoblastic leukemia
BL: Burkitt lymphoma
MCL: Mantle cell lymphoma
FCS: Fetal calf serum
P/S: Penicillin streptomycin
AML: Acute myeloid leukemia
mAb: Monoclonal antibody
PBS: Phosphate buffered saline
SMCC: Sulfonated succinimidyl-4-(N-maleimidomethyl)cyclohexan-1-carboxylate
mAb-P/P: Monoclonal antibody-protamine + free MCC-protamine
LSM: Laser scanning microscopy
RT: Room temperature
FRI: Fluorescence reflectance imaging
PAGE: Polyacrylamide gel electrophoresis
SP-SSC-A: Small particle side-scatter area
SSC-A: Side-scatter area
scrm: Scrambled
ANOVA: Analysis of variance
DNMT3A: DNA (cytosine-5)-methyltransferase 3 A
ADC: Antibody-drug conjugate
EGFR: Epidermal growth factor receptor
IGF1R: Insulin-like-growth factor receptor 1
RNAi: RNA interference
