# Supplementary material for: Transport of cytotoxic cyanine dyes coupled to siRNA into hematopoietic cancer cells with CD20-, CD22- or CD33-targeting ELART nanocarriers
Source: Sci Rep. 2026 Aug 18;16:25877. doi: 10.1038/s41598-026-66482-5 (PMC13486756; doi:10.1038/s41598-026-66482-5)
Supplement: Supplementary file 1 [file 41598_2026_66482_MOESM1_ESM.docx]

SUPPORTING INFORMATION

Transport of cytotoxic cyanine dyes coupled to siRNA into hematopoietic cancer cells with CD20-, CD22- or CD33-targeting ELART nanocarriers

Timo Krassuski ^1,$^, Nicole Bäumer ^1,$^, Stephan Niland ^2^, Katharina Grunert ^1^, Lisa Wittmann ^1^,

Johannes A. Eble ^2^, Georg Lenz ^1^, Wolfgang E. Berdel ^1^, Sebastian Bäumer ^1*^

^1^ Department of Medicine A, Hematology, Oncology and Pneumology, University Hospital of Muenster, Albert-Schweitzer-Campus 1, 48149 Muenster, Germany.

^2^ Institute for Physiological Chemistry and Pathobiochemistry, University of Münster, Waldeyerstraße 15, 48149 Münster, Germany

$ These authors contributed equally.

* Corresponding author

**Supplementary Figures**

**Figure S1: CEX chromatography** **of anti-CD22-mAb-P/P and anti-CD33-mAb-P/P conjugates**. **A and C.** Chromatogram of the CEX chromatography of anti-CD22-mAb-P/P (A) and anti-CD33-mAb-P/P (C). UV absorbance is shown in blue, conductivity in brown and fraction pump B in green. A small peak was evident in the flow-through fraction, representing the unconjugated mAbs, whereas the elution peak exhibited two maxima corresponding to mono- and bi-conjugated mAbs. **B and D.** PAGE analysis of the CEX chromatography of anti-CD22-mAb-P/P (B) and anti-CD33-mAb-P/P (D). In the flow-through fraction, only faint bands for unconjugated mAb were present, whereas prominent bands corresponding to protamine-conjugated mAb were evident in the elution fraction (indicated by a size shift due to protamine addition). Uncropped gel images are given in Figure S2 C.

**Figure S2: Uncropped images of PAGE and agarose gels as shown in Figure 1 and S1.** **A.** Uncropped PAGE gels of lanes shown in Figure 1 B. The relevant lanes shown in the manuscript are indicated by blue arrows. Left: anti-CD20-mAb and anti-CD20-mAb-P/P; Middle: anti-CD22-mAb and anti-CD22-mAb-P/P; Right: anti-CD33-mAb and anti-CD33-mAb-P/P. **B.** Uncropped agarose gels of band shift assays shown in Figure 1C-F. Relevant areas are highlighted by a red box. **C.** Uncropped PAGE gels of CEX fractions as shown in Figure 1 I and Supplementary Figure S1 B and D.

**Figure S3: Size reference beads and proportionality between size and siRNA content of nanocarriers.** **A.** Non-linear fit of the SP-SSC-A geometric means of the size reference beads with the formular y=a+bx^c^. The fit resulted in a R^2^ of 0.9945 with a = 0.08365, b = 0.005211 and c = 0.4856. **B.** Histogram of the SP-SSC-A for the size reference beads. Bead sizes of 0.1, 0.2, 0.5, 1 and 2 µm are shown in a color gradient from red to yellow. Each particle size is well separated from another and results in a distinct population. **C-H.** Flowcytometry analysis of anti-CD20-mAb-P/P (**C**), anti-CD22-mAb-P/P (**D**) and anti-CD33-mAb-P/P (**E**) nanocarriers complexed with Cy5-siRNA. **F-H.** Flow-cytometry analysis of anti-CD20-mAb-P/P (**F**), anti-CD22-mAb-P/P (**G**) and anti-CD33-mAb-P/P (**H**) nanocarriers complexed with Cy3-siRNA. Fluorescence intensity increased proportionally with SSC-A irrespective of the targeting antibody or siRNA label (Cy3 or Cy5). The gate shown separates non-fluorescent particles present in the solvent (red) from nanocarriers with evident fluorescence (blue/green).

**Supporting Methods**

**Cell culture**

All cells were maintained at 37 °C with 5 % CO₂ and high humidity. B-Cell acute lymphoblastic leukemia (B-ALL) cell lines Reh (RRID:CVCL_1650) and 697 (RRID:CVCL_0079), Burkitt lymphoma (BL) cell line Raji (RRID:CVCL_0511) and mantle cell lymphoma (MCL) cell line Mino (RRID:CVCL_1872) were cultured in RPMI-1640 supplemented with 20 % fetal calf serum (FCS), 1 % streptomycin and penicillin (P/S) and 1 % sodium pyruvate (final 1 mM). Acute myeloid leukemia (AML) cell line OCI-AML-2 (RRID:CVCL_1619) was cultured in IMDM supplemented with 20 % FCS and 1 % P/S. For transient expression ExpiCHO-S cells (RRID:CVCL_5J31) were maintained in ExpiCHO-S expression medium and the anti-CD33-mAb producing stable cell line was maintained in ExpiCHO stable production medium (Cat. No. A2910001 and A3711001, respectively, Thermo Fisher Scientific Inc., Waltham, MA, USA). Cells were regularly tested for mycoplasma.

**Expression of anti-CD22 and anti-CD33 monoclonal antibodies (mAbs)**

Anti-CD22 antibody inotuzumab was produced by transient transfection of ExpiCHO-S cells (RRID:CVCL_5J31) using the ExpiCHO expression system kit (Cat. No. A29133, Thermo Scientific Inc., Waltham, MA, USA) according to manufacturer’s instructions. Anti-CD33 antibody gemtuzumab was produced with an ExpiCHO-S cell line generated using the ExpiCHO expression system kit. Supernatant was purified using a CaptureSelect CH1-XL column (Cat. No. 494346201, ThermoFisher Scientific, Waltham MA, USA) and eluted mAbs were then transferred to PBS by gel filtration chromatography using HiPrep 26/10 desalting columns (Cat. No. GE17-5087-02, Sigma-Aldrich, St. Louis, MO, USA). For all chromatography steps an Äkta Pure™ 25 (1.2.0.0) system with data acquisition via UNICORN™ 6.3 (all Cytiva, Washington, D.C., USA) was used.

**Band shift assay**

To determine the binding capacity of the mAb-P/P conjugate a band shift assay was performed. A constant amount of 15 pmol of siRNA (1 µL of 15 µM siRNA in nuclease free water) was complexed with mAb-P/P conjugate in molar antibody to siRNA ratios of 1:1, 1:2, 1:4, 1:8, 1:16 and 1:32 and filled up to 10 µL with PBS. For higher ratios mAb-P/P conjugates were prediluted in PBS to allow pipetting. After 1 h of incubation, 2 µL of 6x DNA loading dye (Cat. No. R0611, ThermoFisher Scientific, Waltham MA, USA) were added and samples were applied to a 2 % agarose (Cat. No. 840001, Biozym, Hessisch Oldendorf, Germany) gel and separated for 15 min at 120 V. For detection of native siRNA ROTI GelStain (Carl ROTH, Karlsruhe, Germany) was added to the gel, whereas no gel stain was used to detect Cy5-labeled siRNA.

**Protein analysis**

For analysis of mAbs and success of mAb-P/P conjugation on Coomassie-stained SDS-PAGE, 20 µL samples were denatured with 2.5 µL reducing agent and 5 µL loading dye (Cat. No. NP0009 and NP0007, respectively, Thermo Fisher Scientific Inc., Waltham, MA, USA) at 95 °C for 5 min and loaded on 15 % SDS-PAGE gels. Coomassie gel staining was performed with Quick Coomassie Stain (Cat. No. GEN-QC-STAIN-1L, Protein Ark, Rotherham, UK) overnight at RT.

**Cation exchange chromatography**

To measure the degree of antibody conjugation a cation exchange chromatography using a Resource S column (Cat. 17117801, Cytiva, Wilmington DE, USA) was performed. For chromatography the conjugate was diluted in PBS (pH 6.5) to a final concentration of 0.75 mg/mL referring to the antibody and loaded onto the column. Elution was then conducted with PBS (pH 6.5) + 2 M NaCl to elute antibody-protamine conjugates and free MCC-protamine. The area under the curve from the flow-through (unconjugated antibody) and elution fraction was used to calculate the conjugation efficacy.

**Flow cytometry analysis**

All flow cytometry measurements were performed with the FACSymphony™ A1 with a small particle detector and FACSDiva software (Becton, Dickinson and Company, Franklin Lakes, NJ, USA). For flow cytometry assessment of internalization, 300 µL of cell suspension were seeded in 48-well plates at 5x10^5^ cells/mL, treated with 60 nM of anti-CD20, anti-CD22 or anti-CD33-mAb-P/P nanocarriers complexed with Cy5-siRNA, and incubated for 1-24 h. Cells were washed with 1 mL PBS, centrifuged, and resuspended in 100 µL PBS in preparation for flow cytometry.

**Flow cytometric size determination of nanocarriers**

For size determination of nanocarriers fluorescent size reference beads (Flow Cytometry Sub-micron Particle Size Reference Kit, Cat. No. F13839, Thermo Fisher Scientific, Waltham, MA, USA) were analyzed and gated to the fluorescent population to exclude nonfluorescent debris. The geometric means of the SP-SSC-A (small particle side scatter area) were used to calculate a non-linear fit with GraphPad Prism version 11.0.2 (GraphPad Software, Boston, MA, USA) following the formular y=a+bx^c. Nanocarriers were assembled as described in the corresponding methods section and diluted with PBS to a concentration of 60 nM in 200 µL, transferred into a FACS-tube and analyzed on a low flow rate for 60 seconds. The geometric mean of the SP-SSC-A was converted to diameter via the abovementioned formular.

**Laser scanning microscopy (LSM)**

For LSM analysis, nanocarriers were assembled as described above, diluted with PBS to a final concentration of 240 nM (in 200 µl), and applied to microscope slides (Starfrost, Cat. No. 11270, Engelbrecht, Edermünde, Germany) for 2 h at RT. Adhered nanocarriers were optionally stained with an anti-human-IgG-Alexa-647 antibody (0,5 µL in 100 µL PBS) for 1 h at RT (Cat. No. 109–607-003, Dianova, Hamburg, Germany), washed with PBS and mounted with DAKO fluorescent mounting medium (Cat. No. S3023, Dako North America, Carpinteria, CA, USA).

To detect the subcellular localization of Cy5-siRNA, cells were seeded at 6x10^4^ cells in 300 µL in 8 well ibiTreat µ-slides (Cat. No. 80826, ibidi, Gräfelfing, Germany) and treated for 24 h with 60 nM nanocarriers containing Cy5-siRNA. At 37 °C, the cells were then stained for 60 min with Lysoview 550 (Cat. No. 70054, Biotium, Fremont, CA, USA; final conc. 2x), 30 min with Mitoview Green (Cat. No. 70083, Biotium, Fremont, CA, USA; final conc. 100 nM) and 10 min with Hoechst 333342. Microscopic examination was performed immediately afterwards by LSM (LSM 800, Zeiss, Oberkochen, Germany).

**Cell titer glow (CTG) assay**

To determine the influence of treatment on proliferation and viability, cells were seeded in 96 well plates at 5x10^4^ cells/well, treated with 60 nM or 240 nM nanocarrier, and treated a second time 24 h after initial treatment. 72 h after initial treatment the CTG assay (Cat. No. G7570, Promega, Madison, WI, USA) was performed according to manufacturer’s instructions. Luminescence was measured on a Victor plate reader (Perkin Elmer, Rodgau, Germany).

**TMRM (tetramethylrhodamine methyl ester) assay**

To measure the mitochondrial membrane potential via TMRM assay, cells were seeded in 96 well plates at 5x10^4^ cells/well, treated with 60 nM or 480 nM nanocarriers and incubated for 48 h. The cells were then diluted in TMRM-containing medium to a final concentration of 20 nM TMRM, incubated at 37 °C for 30 min, centrifuged, washed with 1 mL PBS, and resuspended in 200 µL PBS for flowcytometry analysis.

**Biodistribution**

All animal experiments in this study were carried out in accordance with the recommendations of the state office Animal Care and Use Committee ‘‘Landesamt für Natur, Umwelt und Verbraucherschutz NRW” (LANUV). This study was performed with permission of the Institutional Animal Care and Use Committee and of the local veterinary administration of Muenster (Permit Number 81-02.04.2019.A341). For the generation of a xenograft model, 1x10^7^ OCI-AML2 cells were subcutaneously transplanted into 12-week-old female NU/NU CD1 mice (Charles River Laboratories, Sulzfeld, Germany). Tumor size was frequently analyzed by caliper measurements. At a tumor size of 800-1100 mm^3^, treatment was performed by intraperitoneal injection of anti-CD33-mAb-P/P complexed with fivefold molar excess of DNMT3A-Cy5-siRNA at 4 mg/kg body weight referring to the anti-CD33-mAb (39 µL of 80 µM DNMT3A-Cy5-siRNA with 92.6 µL of anti-CD33-mAb-P/P (6.7 µM), diluted with 18.4 µL of PBS, injection of 150 µL per mouse). Animals were euthanized by CO_2_ inhalation with subsequent cervical dislocation and analyzed 4 or 24 h after treatment. For this experiment, one control mouse treated with PBS and 4 mice treated with anti-CD33-mAb-P/P nanocarriers were used. Organ distribution of Cy5-fluorescent nanocarriers was analyzed *ex vivo* with fluorescence reflectance imaging (FRI, *in vivo* MS FX Pro, Bruker Biospin MRI GmbH, Ettlingen, Germany). To analyze Cy5-fluorescence on a cellular level, tumor sections were passed through a cell strainer (40 µm) into 5 mL PBS. Tumor cells isolated in this manner were washed with PBS and resuspended in 100 µL PBS. For flow cytometry analysis, the cells were further diluted 1:10 in PBS and, optionally, stained for CD33 surface-expression by incubation with an anti-CD33-FITC antibody (Cat. No. 303303, Biolegend, San Diego, CA, USA) for 30 min on ice prior to analysis. Tumors were embedded in cryomatrix (Cat. No. 6769006, Epredia, Kalamanzoo, MI, USA) using standard methods, sectioned, counterstained with Hoechst 33342, mounted with DAKO medium (Cat. No. S3023, Dako North America, Carpinteria, CA, USA) and finally analyzed for Cy5-fluorescence.

**Statistics and Reproducibility**

Statistics were performed with GraphPad Prism software 11.0.1. For TMRM and CTG assays a one-way ANOVA was calculated and if a significant difference (p < 0.05) was detected a Sidak’s multiple comparisons test was calculated comparing PBS with all treatments and comparing all Cy3/Cy5 treatments to respective scrambled siRNA controls. For the TMRM assay three biological replicates were used while for the CTG assay three technical replicates of all three biological replicates were used. Prior to ANOVA a Shapiro Wilk test was conducted to test for normality. All TMRM measurements conform to normality. In a few CTG assay groups the Shapiro Wilk test did not pass normality criteria. Likely this is due to the small sample size and as we observed normality in CTG assays with a larger sample size we still assumed normality for all CTG assays and conducted ANOVA.

**Table 1 ANOVA p-values for TMRM and CTG assays given for each cell line.**

| Cell line | ANOVA p-value TMRM assay | ANOVA p-value CTG Assay |
| --- | --- | --- |
| Raji | <0,0001 | <0,0001 |
| Mino | <0,0001 | 0,0002 |
| Reh | <0,0001 | <0,0001 |
| 697 | <0,0001 | <0,0001 |
| OCI AML2 | <0,0001 | <0,0001 |
